# The Association Between Modified Albumin-Bilirubin (mALBI) and Survival in Advanced Non-small Cell Lung Cancer Patients Treated With Immunotherapy

**DOI:** 10.7759/cureus.56074

**Published:** 2024-03-13

**Authors:** Xiaoya Xu, Xiangru Shi, Dingjie Zhou, Dedong Cao

**Affiliations:** 1 Oncology, People’s Hospital of Macheng City, Macheng, CHN; 2 Oncology, Renmin Hospital of Wuhan University, Wuhan, CHN

**Keywords:** survival, non-small cell lung cancer, prognosis, malbi, immune checkpoint inhibitor

## Abstract

Objective: This study aimed to assess the clinical value of the modified albumin-bilirubin (mALBI) grade in predicting the survival of patients with advanced non-small cell lung cancer (NSCLC) treated with immunotherapy.

Methods: We conducted a retrospective cohort study of patients with advanced NSCLC who received immune checkpoint inhibitors (ICIs) from January 2020 to May 2022. The primary endpoints were overall survival (OS), treatment response, and the association between different mALBI grades and survival.

Results: In these 67 patients, 85.1% (57/67) were male, and the median age was 63 years. The patients with mALBI grades 1 and 2a at baseline had a median OS of 12.83 months (95% CI: 9.4 to 16.27 months), whereas it was 3.2 months (95% CI: NA to 11.59 months) for patients with mALBI grades 2b and 3. The OS for patients with dynamic mALBI grades 1 and 2a was 13.27 months (95% CI: 8.72 to 17.81 months), significantly longer than five months (95% CI: 2.47 to 7.53 months) for dynamic mALBI grades 2b and 3 patients (p<0.01).

Conclusion: In conclusion, mALBI grade may be a potential dynamic biomarker for predicting the prognosis in advanced NSCLC patients treated with immunotherapy.

## Introduction

Lung cancer is one of the most common and deadliest malignancies worldwide [1]. Non-small cell lung cancer (NSCLC) accounts for approximately 85% of 2200000 new diagnoses in 2020, with lung adenocarcinoma and squamous cell lung cancer being the most common histological subtypes, respectively [2,3]. How to cure this disease is still challenging for both patients and oncologists.

A series of driver genes related to NSCLC have been discovered. These genes provide cancer cells with a selective growth advantage, leading to the development of malignant tumors [4]. Targeted drugs designed against driver genes have significantly changed the treatment strategy for advanced NSCLC [5,6]. However, resistance eventually develops to targeted therapy, and how to choose subsequent treatment for driver gene-positive patients is currently a hot topic in clinical research.

In recent years, immune checkpoint inhibitors (ICIs) have made significant breakthroughs in the treatment of driver gene-negative, advanced NSCLC patients, with a five-year survival rate ranging from 13.4% to 23.2% [7,8]. Therefore, ICIs have become the standard treatment for advanced driver gene-negative NSCLC [9]. However, the efficacy of immunotherapy in driver gene-positive patients differs significantly. For example, the efficacy of immunotherapy in patients with epidermal growth factor receptor (EGFR) mutations has not been significantly improved [10]. Numerous clinical studies have demonstrated that a considerable proportion of patients fail to derive benefits from immunotherapy [11-13]. Although immunotherapy has become one of the available treatment options for this population, related efficacy prediction indicators are limited. Finding out how to screen patients who can benefit from immunotherapy is urgent in the clinic. Therefore, a comprehensive analysis of patients with different driver gene statuses who received ICI therapy can help to identify potential predictive indicators of survival.

The albumin-bilirubin (ALBI) grade has emerged as a reliable, objective, and easily accessible indicator for evaluating liver function in recent years. Its high grade indicates poor liver function [14,15]. Therefore, it has been used in multiple studies for prognosis and survival evaluation of malignant tumors such as hepatocellular carcinoma and extrahepatic bile duct carcinoma [14,16,17]. Although the prognostic value of ALBI grading in advanced NSCLC patients has been reported previously, its clinical application is limited due to its continuous features [18]. Besides, the predictive value of modified ALBI (mALBI) grades on survival in advanced NSCLC patients treated with immunotherapy is still not clear.

Therefore, the purpose of this study is to evaluate the predictive value of mALBI grades on the survival of advanced NSCLC patients who received immunotherapy.

## Materials and methods

Patients

A total of 76 patients with advanced NSCLC admitted to the People’s Hospital of Macheng from January 2020 to May 2022 were retrospectively analyzed. Baseline characteristics, treatment details, imaging, and biochemical indicators within one month before and after immunotherapy were collected to evaluate their correlations with survival endpoints.

Inclusion criteria were as follows: (1) pathologically or cytologically diagnosed with NSCLC and confirmed with imaging examinations as an advanced disease; (2) expected survival time >3 months; (3) received at least one cycle of treatment with ICIs; (4) complete baseline data, blood routine, and biochemical tests before and after ICIs treatments; and (5) age >18 years; (6) had imaging-based efficacy evaluation results.

The exclusion criteria were as follows: (1) patients with severe diseases of other organs or systems, such as uncontrolled hypertension; (2) patients who did not receive immunotherapy; (3) patients who claimed he/she experienced mental illness; (4) incomplete clinical data; and (5) patients who could not obtain prognosis or follow-up information.

The study involving human subjects was conducted in accordance with ethical standards and was approved by the Medical Ethics Committee (Institutional Review Board) of the First People’s Hospital of Macheng (Approval Number: 2022-JY005). All procedures performed in the study were in accordance with the ethical standards of the institutional and/or national research committee and with the 1964 Helsinki Declaration and its later amendments or comparable ethical standards. 

Treatments

All enrolled patients received at least one cycle of ICIs, including camrelizumab, pembrolizumab, nivolumab, sintilimab, and other agents. Immunotherapy was continued until patients experienced tumor progression, death, or unacceptable adverse events. When treated with immunotherapy, chemotherapy was administered currently or one day later for some patients. The chemotherapy regimens included paclitaxel in combination with cisplatin or pemetrexed plus cisplatin. Patients received chemotherapy on day 1 of a 21-day cycle. For these regimens, they were used according to the treatment guidelines for lung cancer [19]. Some patients had a history of radiotherapy, which was mainly targeted at the primary lesion in the lung or metastatic lesions.

Endpoints

We collected the baseline characteristics, such as age, sex, Eastern Cooperative Oncology Group performance status (ECOG PS), weight, height, and disease history prior to immunotherapy, as well as treatment responses during immunotherapy treatments. We used overall survival (OS) as the main endpoint and defined OS as the time from the first dose of immunotherapy to death by any causes. Treatment efficacy was assessed by dynamic imaging exams, such as computed tomography or magnetic resonance imaging, according to the Response Evaluation Criteria in Solid Tumors (RECIST) [20]. In addition to OS, progression-free survival (PFS) was also considered as a key endpoint. PFS was defined as the duration from the initiation of immunotherapy until the first documentation of disease progression or death from any cause, whichever occurs first [20]. According to the criteria, tumor lesions that disappeared for more than four weeks are considered complete remission (CR) [20]. Tumors shrinking more than 30% in diameter compared to baseline and maintained for more than four weeks are considered partial remission (PR) [20]. Tumor lesions with a diameter increase of less than 20% or a decrease of less than 30% compared to baseline are considered stable disease (SD) [20]. Tumor lesions with a diameter increase of more than 20% or the appearance of new lesions are considered progression disease (PD) [20]. The objective response rate (ORR) is calculated as the rate of the sum of CR and PR to total participants, and the disease control rate (DCR) is calculated as the sum of CR, PR, and SD rates.

Indicators

The serum samples used in this study were obtained from patients who agreed to undergo routine biochemical tests before the initiation of first-line immune therapy in real-world practice. The main biochemical markers used in this study included serum levels of albumin and total bilirubin at the time of beginning (baseline) and after (dynamic) ICI treatments.

According to previous reports, the calculation formula for ALBI grade is as follows: ALBI score=(\log_{10}{Bilirubin}\times 0.66-0.085\times albumin), where the unit for bilirubin is µmol/L and albumin is g/L [21]. The ALBI and mALBI grades are classified and presented in Table 1 [21]. The neutrophil-lymphocyte ratio (NLR) is calculated as the number of neutrophils divided by the number of lymphocytes (NLR=\frac{neutrophil count}{lymphocyte count} ) [18]. The correlation between ALBI data and treatment outcomes of advanced NSCLC patients receiving immune therapy was analyzed.

**Table 1:** The definition of ALBI and mALBI grades ALBI, albumin-bilirubin; mALBI, modified albumin-bilirubin.

Grade	ALBI Value Range	mALBI Value Range
Grade 1	≤-2.60	≤-2.60
Grade 2	-2.60 to -1.39	2a: >-2.60 and ≤ -2.27
		2b: >-2.27 and ≤-1.39
Grade 3	≥-1.39	≥-1.39

Statistical methods

All statistical analyses were performed using SPSS 23 and R 4.2.2 software. Continuous variables were expressed as mean ± standard deviation or median (interquartile range), while categorical variables were presented as frequency or number (n) and its percentages (%), and compared using the χ2 test or Fisher's exact test. OS was evaluated using Kaplan-Meier analysis, and independent prognostic factors associated with survival were determined using univariate and multivariate Cox regression analysis. The predictive value of various variables was determined using receiver operating characteristic (ROC) curves, and the area under the curve (AUC) of the ROC curve was used to evaluate the accuracy of these predictive factors, including the mALBI grading system, in predicting the OS of patients with advanced NSCLC. A p<0.05 was considered statistically significant.

## Results

Baseline characteristics

**Figure 1:**
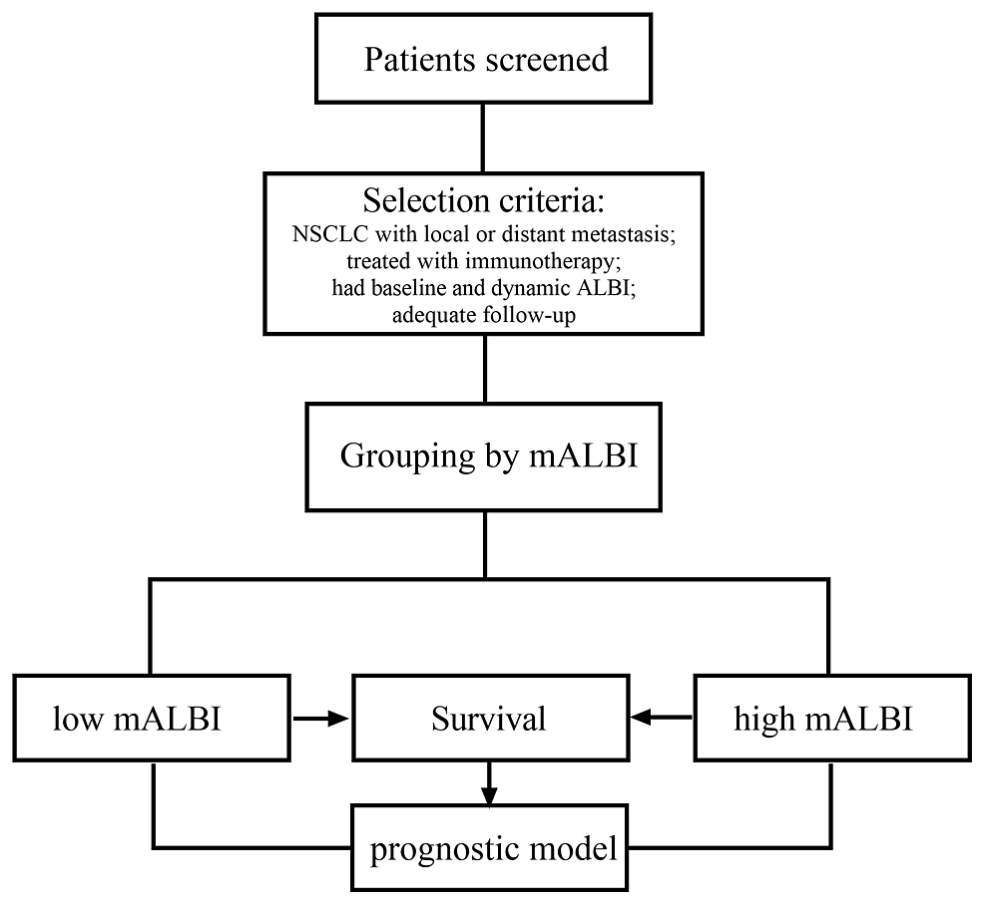
Study flow chart

This study included a cohort of 76 patients diagnosed with advanced NSCLC. Among them, 67 patients (n=67, 100%) satisfied the inclusion criteria and were subsequently monitored for a median period of 12 months. Utilizing the eighth edition of the TNM staging system, it was determined that all participants displayed signs of either local or distant metastasis at the time of enrollment. The study flow is illustrated in Figure 1.

Among the patients, a majority of 51 patients (76.1%) presented with an ECOG PS ranging from zero to one. An additional 14 patients (20.9%) had a PS of two, while two patients (3.0%) were classified with a PS of three. The mean age of the cohort was 62.96 ± 8.87 years (Table 2). The cohort included 57 males (85.1%) and 10 females (14.9%). 

**Table 2:** Demographic and treatment history of the subjects The data has been represented as N, %, or mean ± SD as indicated in the table. ECOG PS, Eastern Cooperative Oncology Group performance status; NA, not available; SD, standard deviation; BMI, body mass index

Variables	Groups	Types	Overall
Number	-	-	67
Sex	Female	%	10 (14.9)
	Male	%	57 (85.1)
Age (years)	-	Mean (SD)	62.96 (8.87)
ECOG PS	-	Mean (SD)	1.27 (0.51)
Height (cm)	-	Mean (SD)	167.93 (5.80)
Weight (Kg)	-	Mean (SD)	60.64 (6.62)
BMI	-	Mean (SD)	21.53 (2.31)
Gene mutation	No or NA	%	61 (90.1)
	Yes	%	6 ( 9.0)
Treatment line	1	%	37 (55.2)
	2	%	20 (29.9)
	3	%	10 (14.9)
Immunotherapy cycles	-	Mean (SD)	4.16 (3.74)
Chemotherapy	No	%	13 (19.4)
	Yes	%	54 (80.6)
Targeted therapy	No	%	61 (91.0)
	Yes	%	6 ( 9.0)
Radiotherapy	No	%	40 (59.7)
	Yes	%	27 (40.3)
Number of metastasis	No	%	13 (19.4)
	1	%	22 (32.8)
	2	%	14 (20.9)
	3	%	6 ( 9.0)
	4	%	9 (13.4)
	5	%	2 ( 3.0)
	≥6	%	1 ( 1.5)

All patients underwent at least one cycle of immunotherapy, either as monotherapy or combined with other treatment modalities. Specifically, 37 patients (55.2%) received immunotherapy as a first-line treatment, whereas 20 patients (29.9%) were treated with immunotherapy in a second-line setting. Chemotherapy was administered to 54 patients (80.6%), and radiotherapy was opted by 27 patients (40.3%).

According to baseline ALBI grades, 39 patients (58.2%) were classified as grade 1, 27 (40.3%) as grade 2, and one (1.5%) as grade 3. Regarding baseline mALBI grades, 39 patients (58.2%) fell into grade 1, 18 (26.9%) into grade 2a, nine (13.4%) into grade 2b, and one (1.5%) into grade 3 (Table 3).

**Table 3:** Baseline and dynamic hematological and biochemical variables The data has been represented as N, %, or mean ± SD as indicated in the table. ALBI, albumin-bilirubin; NLR, neutrophil-to-lymphocyte ratio; mALBI, modified albumin-bilirubin

Variables	Groups	Types	Overall
Baseline neutrophils count (10^9^/L)	-	Mean (SD)	5.01 (2.48)
Baseline lymphocyte count (10^9^/L)	-	Mean (SD)	1.04 (0.45)
Baseline albumin (g/L)	-	Mean (SD)	39.71 (5.37)
Baseline total bilirubin (μmol/L)	-	Mean (SD)	10.71 (5.79)
Baseline NLR	-	Mean (SD)	6.17 (5.61)
Baseline ALBI	-	Mean (SD)	-2.73 (0.47)
Baseline mALBI	1 and 2a	%	56 (83.6)
	2b and 3	%	10 (14.9)
	NA	%	1 (1.5)
Dynamic neutrophils count (10^9^/L)	-	Mean (SD)	4.83 (3.72)
Dynamic lymphocyte count (10^9^/L)	-	Mean (SD)	0.93 (0.47)
Dynamic albumin (g/L)	-	Mean (SD)	38.77 (6.04)
Dynamic total bilirubin (μmol/L)	-	Mean (SD)	9.12 (5.37)
Dynamic NLR	-	Mean (SD)	6.46 (5.92)
Dynamic ALBI	-	Mean (SD)	-2.70 (0.56)
Dynamic mALBI	1 and 2a	%	52 (77.6)
	2b and 3	%	11 (16.4)
	NA	%	4 (6.0)
Dynamic - baseline ALBI	-	Mean (SD)	0.07 (0.46)

According to the baseline ALBI grades, 39 patients (58.2%) were categorized as grade 1, 27 patients (40.3%) were assigned to grade 2, and one patient (1.5%) was classified as grade 3. Similarly, when evaluated using the mALBI grading system, 39 patients (58.2%) were graded as 1, 18 patients (26.9%) as 2a, nine patients (13.4%) as 2b, and one patient (1.5%) as grade 3, further detailing the liver function status across the cohort.

Overall efficacy and survival

For the entire cohort (Table 4), the median OS for these cases was 17.8 months (95% CI: 13.32-22.28). Similarly, the median PFS was 9.35 months (95% CI: 6.91-11.79). Within our cohort, treatment outcomes included one patient (1.5%) achieving CR, five patients (7.5%) achieving PR, 45 patients (67.1%) maintaining SD, and 16 patients (23.9%) experiencing PD.

**Table 4:** Treatment efficacy and survival The data has been represented as N, %, or mean ± SD as indicated in the table. PFS, progression free survival; OS, overall survival; CI, confidence interval; CR, complete response; PR, partial response; SD, stable disease; PD, progressive disease

Variables	Groups	Types	Overall
PFS (months)	-	Median and 95% CI	9.35 (6.91-11.79)
OS (months)	-	Median and 95% CI	17.80 (13.32-22.28)
Survival	Alive	%	36 (53.7)
	Dead	%	31 (46.3)
CR	Yes	%	1 (1.5)
PR	Yes	%	5 (7.5)
SD	Yes	%	45 (67.2)
PD	Yes	%	16 (23.9)

Analysis of prognostic factors

To explore potential prognostic factors influencing OS, univariate analysis was conducted, focusing on variables with P<0.2 and key baseline characteristics such as sex for further evaluation in multivariate Cox regression analysis. This preliminary analysis identified several factors, including the number of immunotherapy cycles, baseline NLR, baseline mALBI grade, and dynamic changes in mALBI grade, as potential predictors of OS outcomes (Table 5). 

**Table 5:** Univariate analysis for OS The data are presented as an HR with its related 95% CI. It is considered significant if the p-value is less than 0.05. *Variables with statistical significance ECOG PS, Eastern Cooperative Oncology Group performance status; ALBI, albumin-bilirubin; NLR, neutrophil-to-lymphocyte ratio; mALBI, modified albumin-bilirubin; OS, overall survival; CI, confidence interval; HR, hazard ratio

Variables	P-value	HR	95% CI
Sex	0.41	0.68	0.28-1.7
Age	0.96	1	0.5-2.1
ECOG PS	0.28	1.4	0.75-2.7
Treatment line	0.82	1.1	0.63-1.8
Immunotherapy cycles	0.11	0.52	0.23-1.2
Chemotherapy	0.3	0.62	0.25-1.5
Targeted therapy	0.24	1.9	0.65-5.4
Radiotherapy	0.37	0.71	0.34-1.5
Number of metastasis	0.26	1.1	0.91-1.4
Progressive disease*	2.30E-06	6	2.9-13
Baseline neutrophils count*	0.00029	1.3	1.1-1.5
Baseline lymphocytes count	0.5	0.74	0.3-1.8
Baseline NLR*	0.0065	1.1	1-1.1
Baseline albumin	0.37	0.97	0.9-1
Baseline total bilirubin	0.2	1	0.98-1.1
Baseline ALBI	0.24	1.7	0.72-3.9
Baseline mALBI	0.19	1.6	0.79-3.3
Dynamic mALBI	0.11	1.5	0.92-2.3
Baseline mALBI 1 and 2a	0.099	2.1	0.87-5.2
Dynamic mALBI 1 and 2a*	0.0074	3.1	1.4-7.2
Dynamic - baseline ALBI*	0.018	1.9	1.1-3.1

We then conducted a multivariate Cox regression analysis to ascertain the independent predictors of OS. As shown in Table 6, baseline NLR (p=0.002, hazard ratio (HR)=1.27), number of metastasis (p=0.02, HR=2.64), or dynamic ALBI grades (p=0.03, HR=0.36) were independent predictors of OS.

**Table 6:** Multivariate analysis for OS The data are presented as HR with its related 95% CI. It is considered significant if the p-value is less than 0.05. *Variables with statistical significance. NLR, neutrophil-to-lymphocyte ratio; mALBI, modified albumin-bilirubin; OS, overall survival; CI, confidence interval; HR, hazard ratio

Variables	Comparison	P-value	HR	95% CI
Sex	Female vs. Male	0.174	1.989	0.739-5.353
Radiotherapy	Yes vs. No	0.362	0.677	0.293-1.566
Number of metastasis*	>1 vs. ≤1	0.02	2.637	1.163-5.978
Baseline NLR*	continuous	0.002	1.272	1.096-1.476
Dynamic mALBI*	(1 and 2a) vs. (2b and 3)	0.031	0.361	0.143-0.91

**Figure 2:**
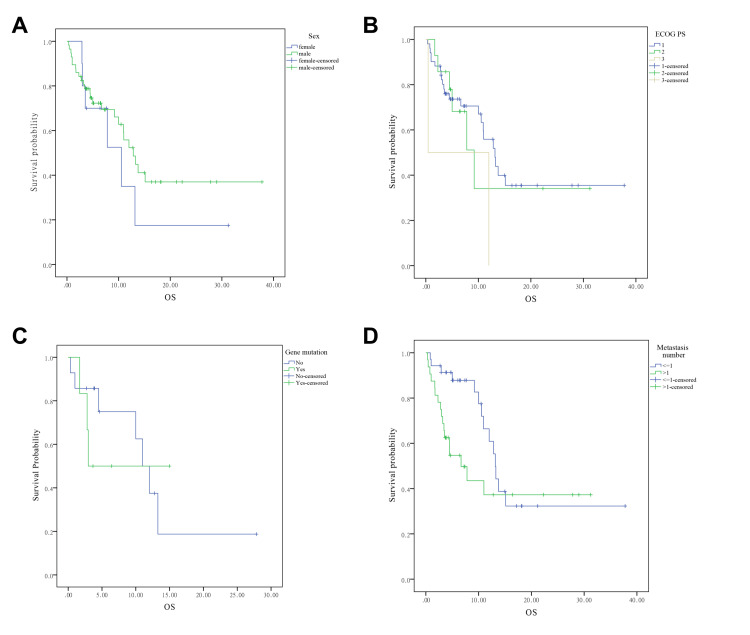
The survival analysis based on disease features in advanced NSCLC patients treated with immunotherapy (A) sex, (B) ECOG PS, (C) gene mutation, and (D) number of metastasis. Data are compared using the log-rank (Mantel-Cox) analysis. A p-value <0.05 is considered statistically significant. NSCLC, non-small cell lung cancer; ECOG PS, Eastern Cooperative Oncology Group performance status; OS, overall survival

Subgroup analyses were performed to assess the influence of various baseline and treatment-related factors on OS. As revealed by the Kaplan-Meier curves (Figure 2), the sex was not significantly associated with OS (Figure 2A, p=0.40), the OS in patients with PS 1-3 (Figure 2B), gene mutation positive and negative (Figure 2C), number of metastasis (Figure 2D, ≤1 vs. >1) were similar (p=0.30, p=0.75, and p=0.08, respectively).

**Figure 3:**
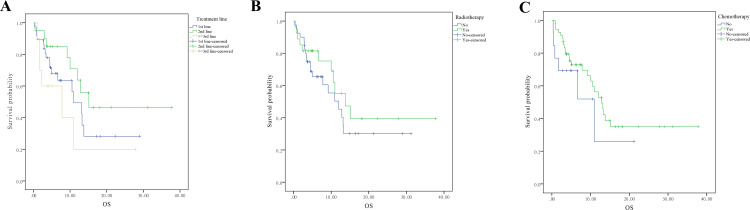
The survival analysis based on treatment regimen in advanced NSCLC patients treated with immunotherapy (A) Treatment line of immunotherapy, (B) radiotherapy, and (C) chemotherapy. Data are compared using the log-rank (Mantel-Cox) analysis. It is considered significant if the P-value is less than 0.05. NSCLC, non-small cell lung cancer

Further analyses indicated that the line of treatment with ICIs did not significantly affect OS (Figure 3A, p=0.15), nor did the history of radiotherapy (Figure 3B, p=0.36) or chemotherapy (Figure 3C, p=0.29) among patients treated with ICIs.

Effect of mALBI on survival of advanced NSCLC after ICI treatment

**Figure 4:**
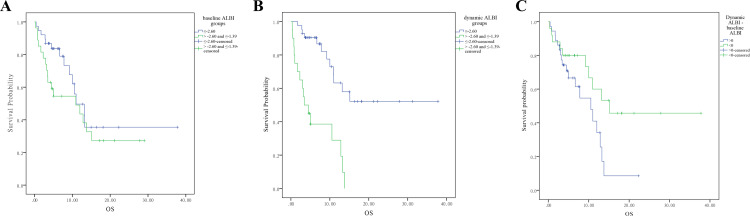
The survival analysis based on ALBI levels in advanced NSCLC patients treated with immunotherapy (A) Baseline ALBI, (B) dynamic ALBI, and (C) ALBI changes calculated from dynamic-baseline ALBI. Data are compared using the log-rank (Mantel-Cox) analysis. It is considered significant if p-value is less than 0.05. NSCLC, non-small cell lung cancer; ALBI, albumin-bilirubin

Our study also assessed the association between ALBI scores and OS. Kaplan-Meier curves showed no significant difference in OS between patients with high versus low baseline ALBI grades (Figure 4A, p=0.19). However, dynamic ALBI scores post-ICI treatment significantly correlated with OS (Figure 4B, p<0.01), with patients having ALBI scores ≤-2.60 showing markedly improved OS compared to those with scores >-2.60 and ≤-1.39. Analysis of changes in ALBI scores from baseline to post-treatment indicated that patients experiencing improvements in ALBI values demonstrated significantly better OS than those with increased ALBI values (Figure 4C, p=0.04).

**Figure 5:**
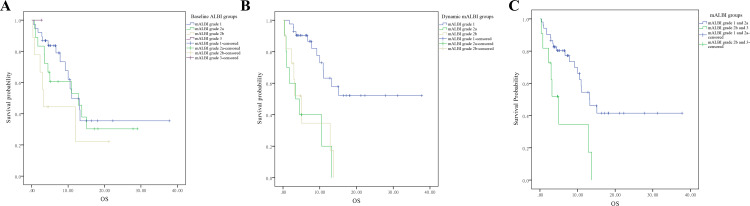
The survival analysis based on disease features in advanced NSCLC patients treated with immunotherapy (A) Baseline mALBI, (B) dynamic mALBI, and (C) groups of dynamic mALBI (1 and 2a) and (2b and 3). Data are compared using the log-rank (Mantel-Cox) analysis. It is considered significant if p-value is less than 0.05. NSCLC, non-small cell lung cancer; mALBI, modified albumin-bilirubin

Regarding the mALBI score, the median OS of patients across baseline mALBI grades 1, 2a, 2b, and 3 did not show significant differences (Figure 5A, p=0.28), while the OS of dynamic mALBI grades 1, 2a, and 2b of patients were 24.1 (95% CI: 18.2 to 30.0), 5.9 (95% CI: 2.5 to 9.4), and 6.4 (95% CI: 2.7 to 10.2) months (Figure 5B, p<0.01), respectively. We conducted subgroup analyses based on dynamic mALBI grades, comparing grades 1 and 2a against grades 2b and 3. Conversely, dynamic mALBI grades showed significant variations in OS, with significant differences noted when comparing lower mALBI grades (1 and 2a) against higher grades (2b and 3) post-ICI treatment (Figure 5C, p=0.005). 

Predictive model based on mALBI

**Figure 6:**
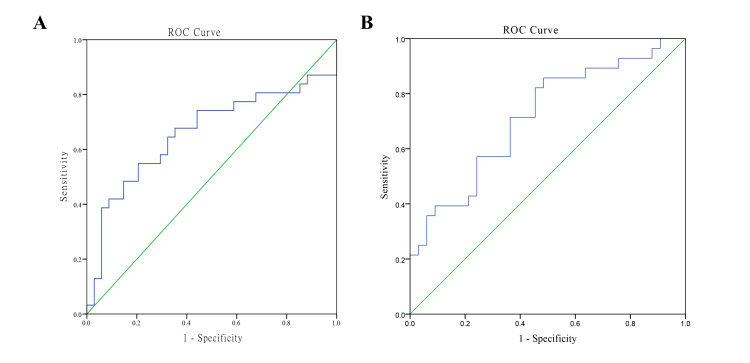
The predictive models for survival based on mALBI grades (A) Baseline mALBI-based model and (B) dynamic mALBI-based model. Data are compared using the AUC analysis. It is considered significant if p-value is less than 0.05. AUC, area under the curve

ROC curve analysis revealed that the mALBI classification model was more predictive of OS than the ALBI classification model (Figure 6). While the baseline mALBI model (Figure 6A) had an AUC of 0.66 (95% CI: 0.52-0.80; p=0.03), the dynamic mALBI model (Figure 6B) showed a higher AUC of 0.72 (95% CI: 0.59-0.85; p=0.004), indicating a slight advantage in predicting OS, with a Youden index of 0.37, sensitivity of 0.86, and specificity of 0.52.

## Discussion

The long-term prognosis of malignant tumor patients is closely related to liver function. Child-Pugh classification is a common indicator for assessing liver function, but it is based on multiple detection indicators and is easily influenced by several subjective factors. Compared with the Child-Pugh classification, the ALBI classification is more objective and easier to obtain. In a previous study, we reported the prognostic value of ALBI in advanced NSCLC patients. However, the disadvantages of ALBI grades limit its clinical utilization. Using the modified ALBI classification method, we conducted this study to further analyze and compare the predictive ability of the mALBI grading system for survival in advanced NSCLC patients, thereby providing a more objective, simple, and effective predictive indicator. In this study, we found that mALBI after immunotherapy was associated with increased survival in advanced NSCLC patients. The improvement of ALBI was also an indicator of better survival in these patients.

For a long time, predicting the clinical efficacy and prognosis of patients receiving immunotherapy has been an important clinical issue. Several studies have explored how to predict efficacy in advanced NSCLC patients receiving ICIs. PD-L1 has shown excellent performance in predicting its efficacy, but its detection relies on tissue specimens, which has been one of the reasons limiting its application. Tumor mutational burden (TMB) has also shown good predictive value for efficacy in advanced NSCLC patients receiving ICIs. However, the high cost of TMB also limits its clinical application. ALBI has demonstrated good prognostic significance in hepatocellular carcinoma and NSCLC. The ALBI grade proposed by Johnson et al. in 2015 is a simple and objective liver function evaluation method based only on serum albumin and bilirubin [14]. It shows reliable prognostic ability in numerous studies and can stratify patients with significantly different prognoses. Pinato et al.'s large multicenter clinical study shows that ALBI grade can serve as an important prognostic predictor of OS in liver cancer patients receiving surgical resection, TACE, and sorafenib treatment [22]. Thus, it may be an effective prognostic indicator for liver cancer. Inevitably, the limitation of ALBI grading is that the serum albumin level may change when patients receive albumin replacement therapy or branched-chain amino acid drugs. Additionally, in patients with relatively good liver function and obstructive jaundice, the serum bilirubin level is usually higher, which affects the accuracy of ALBI grading and may not always reflect the patient's actual liver function. Therefore, in 2017, Hiraoka et al. proposed the mALBI, which divides ALBI grade 2 into 2a and 2b (mALBI level) based on the critical value of "-2.270" (ALBI grade) [23]. Our study found that although baseline mALBI was not significantly associated with OS, dynamic mALBI could still be highly effective in predicting the survival of advanced NSCLC patients after immunotherapy. Moreover, the mean OS in patients with low-grade versus high-grade baseline mALBI was 18.1 versus 8.8 months (p=0.09), respectively. This insignificance may be due to the limited number of included patients and the follow-up duration.

In recent years, research on advanced NSCLC patients with driver gene positivity has found that the status of driver genes is closely related to the efficacy of immunotherapy. Mazieres et al. include 551 patients with driver gene positivity (including KRAS, EGFR, ERBB2, ALK, ROS-1, BRAF, RET) NSCLC, and 94.6% of patients received ICIs monotherapy after TKIs or chemotherapy progression [24]. The results showed that NSCLC patients with driver gene mutations receiving ICI monotherapy had limited survival benefits, with a median PFS of 2.8 months and a median OS of 13.3 months [24]. Borghaei et al. included 82 EGFR mutation NSCLC patients who progressed on EGFR-TKIs or platinum-containing regimens, and nivolumab did not significantly prolong PFS [25]. Rittmeyer et al. included 85 EGFR mutation patients, and atezolizumab did not provide survival benefits to patients [26]. Herbst et al. included 85 EGFR mutation patients, and subgroup analysis showed that pembrolizumab had no survival benefit in this population [27]. A meta-analysis based on several clinical studies confirmed the limited efficacy of ICI monotherapy in the EGFR mutation population [28]. This study included patients with driver gene mutations, who received immunotherapy in the setting of subsequent line treatments, with ICIs combined with chemotherapy being the most common regimen. The median OS of these patients was three months, while that of patients without mutations was 11 months (p=0.75). Furthermore, subgroup analysis using ALBI revealed that regardless of the presence of driver gene mutations, mALBI still maintained good prognostic performance in these patients.

Advanced NSCLC patients often receive immunotherapy along with other anti-tumor therapies, such as radiotherapy and chemotherapy. In this study, subgroup analysis was conducted based on whether they received radiotherapy and/or chemotherapy, and it was found that both radiotherapy and chemotherapy did not significantly affect OS. While patients receiving chemotherapy had a trend of longer OS than those who did not receive chemotherapy, it was not statistically significant (p<0.05). Further analysis using baseline mALBI showed that patients with low mALBI grades could benefit from either radiotherapy or chemotherapy. This suggests that patients with poor liver function are less likely to benefit from high-intensity anti-tumor therapy.

There are several limitations to this study. First, the number of patients included in the study was small and all from a single center. Future multicenter studies are needed to further validate these results. Second, this study is a retrospective analysis and has a certain risk of selection bias. Third, there were heterogeneities in baseline characteristics of the patients, such as some patients having driver gene mutations, which could also affect the effectiveness of immunotherapy. Nevertheless, our study demonstrated that dynamic mALBI is associated with survival in advanced NSCLC patients treated with ICIs.

## Conclusions

In conclusion, this study demonstrates that mALBI is related to the survival of advanced NSCLC patients and shows its potential predictive role in these patients after immunotherapy. Further prospective studies on the mALBI grading system are necessary to validate these findings.
